# Complete remission in an advanced hepatocellular carcinoma patient with AXIN1 mutation after systemic therapy: A case report

**DOI:** 10.1016/j.heliyon.2025.e42010

**Published:** 2025-01-16

**Authors:** Yihong Ran, Xiaozhun Huang, Xu Che, Dong Chen

**Affiliations:** National Cancer Center, National Clinical Research Center for Cancer, Cancer Hospital & Shenzhen Hospital, Chinese Academy of Medical Sciences and Peking Union Medical College, Shenzhen, 518116, China

**Keywords:** Hepatocellular carcinoma, Systemic therapy, Liver biopsy, Hepatic arterial infusion chemotherapy, Clinical complete remission

## Abstract

Primary hepatocellular carcinoma (HCC) is a common malignancy with high morbidity and mortality. Despite progress in systemic therapies, survival in advanced HCC remains poor due to patient heterogeneity and individual differences, necessitating a personalized approach rather than relying solely on guidelines. Here, we present an exceptional case study in which a systematic regimen without immune checkpoint inhibitors was chosen based on the patient's specific genetic test results. Remarkably effective with long-term survival benefits were observed as a result. This case underscores the importance of incorporating tumor profiling and personalized treatment plans, in addition to adhering to guidelines and standards, for delivering more efficacious and well-tolerated therapeutic options to patients with liver cancer.

## 1 Introduction

HCC is a prevalent malignancy worldwide with a dismal prognosis and ranks as the fourth leading cause of cancer-related mortality globally [1]. In Asia, the incidence of HCC is significantly higher compared to other continents, primarily attributed to factors such as hepatitis virus infection, regional dietary patterns, and environmental influences [2]. Even worse, due to the absence of typical initial symptoms, most patients are diagnosed at an advanced stage and lose the chance for surgical intervention [3]. Currently, interventional therapy, radiotherapy, chemotherapy, targeted therapy and immunotherapy constitute the mainstay treatment options for advanced liver cancer [[4], [5], [6]].

In recent years, significant advancements in clinical trials have led to a dramatic shift the systemic therapy for HCC. Traditional chemo and interventional therapies have been partly superseded. Targeted drugs like EGFR and VEGF inhibitors, have demonstrated remarkable efficacy in specific patient populations [6]. In addition, various immune-based agents ranging from PD1/PD-L1 inhibitors to emerging CTLA4 inhibitors have continued to gain approval for liver cancer treatment and have showed promising therapeutic outcomes [7]. This trend underscores the growing recognition of the clinical significance of immunotherapy during the emergent stage of hepatocellular carcinoma [8].

However, the underlying dilemma has not been solved: the overall survival rate of liver cancer remains sub-optimal, especially in advanced cases. This can primarily be attributed to the heterogeneity and individual variances observed in liver cancer [9], as each patient exhibits distinct responses and tolerances towards different treatment modalities. Therefore, personalized approaches should be adopted to maximize both survival rates and quality of life [10].

In this context, we present a unique case of HCC. Based on the patient's specific genetic test results, we opted for a systemic treatment regimen that excluded immune checkpoint inhibitors. Despite deviating from the current mainstream treatment paradigm, remarkable efficacy and long-term survival were achieved. This case underscores the importance of not solely relying on guidelines and norms in diagnosing and treating HCC patients but also leveraging tumor characteristics analysis and individualized treatment plans to offer more effective and tolerable therapeutic options.

## 2 Case presentation

**Fig. 1:**
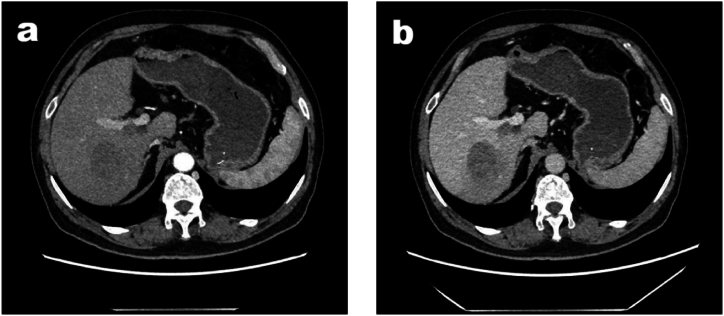
(a). Enhanced CT (arterial phase) showed that the tumor was located in the right posterior lobe with uneven enhancement. Fig. 1b. Enhanced CT (venous phase) showed that the tumor was further enhanced in the venous phase, and the right branch of the portal vein was invaded.

**Fig. 2:**
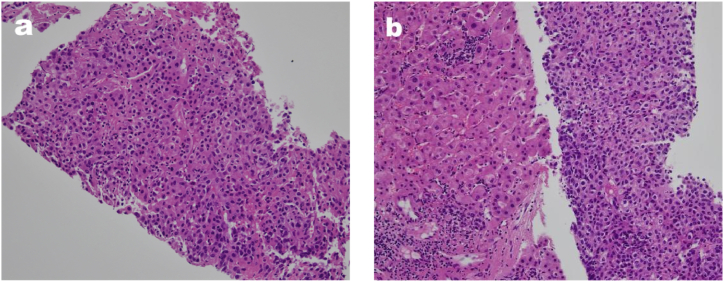
(a and b). The specimen shows cancerous cells manifest in beam-like and nest-like formations, displaying diverse sizes and morphologies. (Hematoxylin and eosin, x100).

The patient, a 67-year-old man with no prior history of hepatitis B, underwent chest, abdomen, and pelvis CT as well as upper abdominal MRI scans on July 23, 2021 at our hospital due to abdominal pain. The imaging examination revealed the presence of a tumor measuring approximately 4.9∗3.8∗4.0cm in the right posterior lobe of the liver, accompanied by invasion of the right branch of the portal vein (Fig. 1a and b). No evidence of metastasis was observed in other organs and lymph nodes, while serum alpha-fetoprotein (AFP) levels were elevated to 3398 ng/mL. Given the patient's clinical history and imaging findings, a definitive diagnosis could not be established. Therefore, we believe that a biopsy is necessary for further evaluation and treatment planning. On July 30, 2021, a fine-needle aspiration biopsy of the liver tumor was performed. Pathological examination confirmed infiltration by poorly differentiated cancer cells into liver tissue samples obtained from biopsy (Fig. 2a and b). Additional immunohistochemical staining results supported a diagnosis consistent with hepatocellular carcinoma with minimal necrosis. Positive staining for CK18 (3+), Hepatocyte antigen (3+), AFP (+), Arg-1(2+), CD34 (+), and Ki67 labeling index (∼40 % hot zone) further supported this diagnosis. Conversely, negative staining was observed for GPC3 (−), CA19-9 (−), CK19 (−), and CK7 (−). According to the AJCC Cancer Staging System Version 8, this case is classified as pT4N0M0 stage HCC [11].

The patient underwent genetic testing after biopsy, revealing an AXIN1 mutation. Previous clinical data indicating that patients with HCC with mutations predicted to result in WNT pathway activation are innately resistant to immune checkpoint blockade. all patients with tumors harboring activating CTNNB1 or inactivating AXIN1 mutations had progressive disease [12]. In addition, patients with the above genetic mutations exhibited shorter progression-free survival compared to those without mutations. Moreover, immunohistochemical analysis showed no expression of PD-L1 in the patient's tumor tissue. Based on these findings, immunotherapy may not provide benefits for this particular patient. Therefore, alternative treatment options including interventional therapy, targeted therapy, and active supportive care were pursued.

**Fig. 3:**
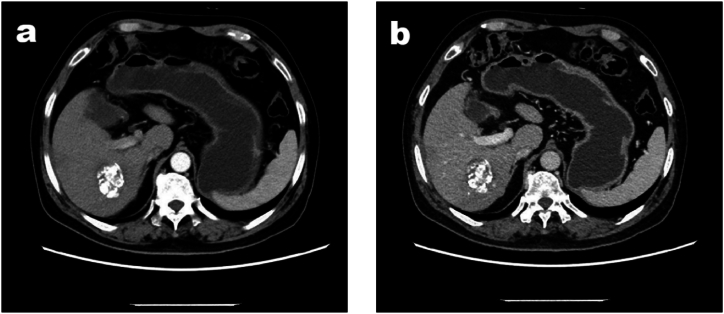
(a). Enhanced CT (arterial stage) showed that the tumor was located in the right posterior lobe, with internal iodol deposits and no enhancement. Fig. 3b. Enhanced CT (venous phase) showed that the tumor was located in the right posterior lobe, with internal iodol deposits.

**Fig. 4:**
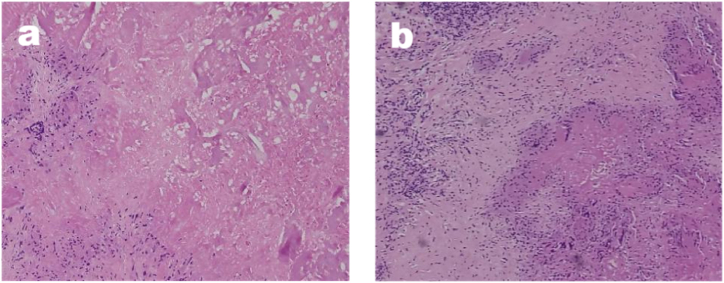
(a and b). The specimen shows no evidence of cancer, but cell necrosis, accompanied by infiltration of inflammatory cells (Hematoxylin and eosin, x100).

Between August 10, 2021 and September 13, 2021, the patient underwent two cycles of transarterial chemoembolization with hepatic arterial infusion of Oxaliplatin, Fluorouracil (TACE/HAIC), followed by initiation of oral Lenvatinib therapy at a daily dose of 12mg. On October 26, 2021, chest, abdomen, and pelvis enhanced CT revealed a significant reduction in liver tumor size and inactivity (Fig. 3a and b), while AFP levels decreased to 49.94ng/ml. During interventional therapy, the patient experienced intolerable pain in the right upper quadrant of the abdomen accompanied by chest tightness, nausea and vomiting leading to discontinuation of interventional therapy. Therefore, a pathological biopsy is essential for assessing the activity of residual liver tumor and providing guidance for subsequent treatment. Subsequent histological examination showed that no malignant cells remained (Fig. 4a and b). The tumor response was classified as pathological complete remission (PCR). We recommend further evaluation by PET/CT examination. However, due to financial constraints, the patient declined this assessment. Consequently, the patient maintained oral administration of Levastinib while discontinuing interventional therapy. During the maintenance treatment phase, a series of adverse reactions emerged. Gingival bleeding was observed, which manifested as sporadic bleeding from the gum tissues, potentially affecting the patient's oral health and causing discomfort. Hypertension also developed, with elevated blood pressure levels that required close monitoring and appropriate management to prevent potential cardiovascular complications. Additionally, hand-foot syndrome presented, characterized by symptoms such as redness, swelling, pain, and paresthesia in the hands and feet, which could have a significant impact on the patient's daily activities and quality of life. However, through the implementation of symptomatic management strategies, which encompassed a range of measures including but not limited to the use of specific medications to control blood pressure, topical agents or oral medications to address gingival bleeding, and palliative care for hand-foot syndrome, these adverse reactions were effectively alleviated. The symptomatic management not only mitigated the severity of the symptoms but also contributed to the patient's ability to tolerate the maintenance treatment, ensuring the continuity of the therapeutic regimen and potentially optimizing the overall treatment outcome.

**Fig. 5:**
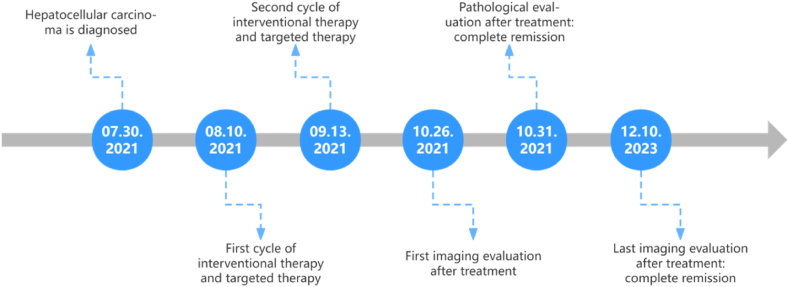
Treatment timeline of this case.

At present, the patient attains complete remission (CR) following systemic therapy, while exhibiting a survival time of more than two years. The systemic treatment regimen, excluding immunotherapy, has showed favorable therapeutic outcomes, and the patient continues to receive this treatment with sustained benefits and minimal treatment-related adverse reactions. A schematic representation of the treatment timeline is presented below(Fig. 5).

## 3 Discussion

Selection of first-line treatment for patients with advanced HCC requires consideration of multiple factors, including tumor oncology characteristics, stage, liver function, and performance status. Systemic treatments such as immunotherapy, chemotherapy, targeted therapy, radiotherapy, and local ablation constitute the primary modalities [13,14]. Ongoing advances in immunotherapy research have provided mounting evidence supporting its combination with traditional treatments to yield enhanced therapeutic efficacy [15]. Therefore, when formulating treatment plans, it is imperative to thoroughly consider patient-specific conditions alongside the latest research progress to provide optimal treatment options.

In recent years, the emergence of immune checkpoint inhibitors has revolutionized the treatment landscape for hepatocellular carcinoma, offering new avenues for precision therapy and promising outcomes. The combination regimens of “Atezolizumab combined with Bevacizumab” and “Durvalumab combined with Tremelimumab” have demonstrated enhanced prognostic outcomes among HCC patients [16,17]. Essentially, immunotherapy harnesses the patient's own immune system to combat cancer cells, potentially leading to reduced side effects and prolonged efficacy compared to conventional agents [18].

In clinical practice, although immunotherapy has showed potential in the treatment of hepatocellular carcinoma (HCC) [19], the management of liver cancer still faces challenges. Notably, HCC exhibits significant heterogeneity, which may contribute to variable responses to immunotherapy among individuals. The factors that influence the decision-making process and the outcome of a decision-making scenario are complex and multifaceted. Taking this patient as an illustrative example, genetic testing revealed a mutation in AXIN1, suggesting a potential limited response to immunotherapy [12]. AXIN1 mutations can lead to abnormal expression of immune checkpoint molecules (such as PD-L1) on the surface of tumor cells. This abnormal expression may not be induced through traditional immune activation pathways but is related to alterations in the Wnt/β-catenin signaling pathway. In such a situation, using immune checkpoint inhibitors may not be able to effectively block immune escape, as tumor cells may maintain an immunosuppressive state through other mechanisms. In light of our commitment to precise tumor therapy, we opted for a systematic treatment approach devoid of immunotherapy and ultimately achieved favorable therapeutic outcomes.

Although the latest liver cancer guidelines have not yet identified the role of micro-satellite instability (MSI), mismatch repair (MMR), tumor mutation burden (TMB), or programmed death ligated 1(PD-L1) in HCC [20], partial immune checkpoint suppression has demonstrated clinical benefit. In fact, there is currently no consensus on whether molecular genetic testing should be conducted for HCC patients. Given the increasing emphasis on individualized tumor therapy, appropriate genetic testing is considered necessary for comprehensive tumor assessment and subsequent treatment plan selection [21]. With advances in precision medicine [22], clinical treatment strategies are shifting from population-based approaches to individualized treatments tailored even to a single tumor.

## 4 Conclusion

Patients with liver cancer harboring AXIN1 mutation may potentially derive therapeutic benefits from an alternative systemic treatment regimen devoid of immunotherapy.

## CRediT authorship contribution statement

**Yihong Ran:** Writing – original draft, Data curation, Conceptualization. **Xiaozhun Huang:** Writing – original draft. **Xu Che:** Writing – review & editing, Supervision. **Dong Chen:** Writing – review & editing, Supervision.

## Informed consent statement

The written informed consent of the patient has been obtained for this case report. Before reporting this case, we explained to the patient in detail the purpose, process, potential risks, and possible benefits of the research. The patient fully understood and voluntarily signed the informed consent form, agreeing to use his/her personal case data (including but not limited to medical history, test results, treatment process, and other information) for medical research and related reporting. This is to promote the dissemination and exchange of medical knowledge and provide a reference example for research in the field of liver cancer treatment. The patient is aware that his/her personal information will be properly handled and protected in accordance with relevant laws, regulations, and ethical norms to ensure that his/her privacy is not disclosed.

## Patient's perspective on their treatments

According to my Performance Status and genetic test results, the doctor devised a systemic treatment plan incorporating interventional therapy and targeted therapy. Following two treatment cycles, reexamination revealed significant efficacy. However, due to physical constraints, adverse reactions arose during interventional therapy, leading me to question my ability to adhere to subsequent sessions. Consequently, I opted solely for targeted therapy which still yielded optimal therapeutic outcomes.

## Data and code availability statement

Data included in the article/supplementary material is referenced in the article.

## Ethics statement

This manuscript follows the International Committee of Medical Journal Editors (ICMJE) recommendations for the conduct, reporting, editing and publication of scholarly work in medical journals. No potential conflicts of interest were disclosed.

## Funding

This work was supported by 10.13039/501100012151Sanming Project of Medicine in Shenzhen (No. SZSM202011010).

## Declaration of competing interest

The authors declare the following financial interests/personal relationships which may be considered as potential competing interests:Xu Che reports financial support was provided by 10.13039/501100012151Sanming Project of Medicine in Shenzhen. Reports a relationship with that includes has patent pending to. If there are other authors, they declare that they have no known competing financial interests or personal relationships that could have appeared to influence the work reported in this paper.

## References

[1] Lindblad K.E., Ruiz De Galarreta M., Lujambio A. (2021). Tumor-intrinsic Mechanisms regulating immune exclusion in liver cancers. Front. Immunol..

[2] Kao J.H. (2014). Risk stratification of HBV infection in Asia-Pacific region. Clin. Mol. Hepatol..

[3] Wang Z., Zhang G., Wu J. (2013). Adjuvant therapy for hepatocellular carcinoma: current situation and prospect. Drug Discov. Therap..

[4] Chen S., Xu B., Wu Z. (2021). Pembrolizumab plus lenvatinib with or without hepatic arterial infusion chemotherapy in selected populations of patients with treatment-naive unresectable hepatocellular carcinoma exhibiting PD-L1 staining: a multicenter retrospective study. BMC Cancer.

[5] Peng Z., Fan W., Zhu B. (2023). Lenvatinib combined with transarterial chemoembolization as first-line treatment for advanced hepatocellular carcinoma: a phase III, randomized clinical trial (launch). J. Clin. Oncol..

[6] Al-Salama Z.T., Syed Y.Y., Scott L.J. (2019). Lenvatinib: a review in hepatocellular carcinoma. Drugs.

[7] Cao L., Zhu L., Cheng L. (2022). ncRNA-regulated layn serves as a prognostic biomarker and correlates with immune cell infiltration in hepatocellular carcinoma: a bioinformatics analysis. BioMed Res. Int..

[8] Sangro B., Sarobe P., HerváS-Stubbs S. (2021). Advances in immunotherapy for hepatocellular carcinoma. Nat. Rev. Gastroenterol. Hepatol..

[9] English K., Brodin N.P., Shankar V. (2020). Association of addition of ablative therapy following transarterial chemoembolization with survival rates in patients with hepatocellular carcinoma. JAMA Netw. Open.

[10] Miki D., Ochi H., Hayes C.N. (2012). Hepatocellular carcinoma: towards personalized medicine. Cancer Sci..

[11] Kamarajah S.K., Frankel T.L., Sonnenday C. (2018). Critical evaluation of the American joint commission on cancer (AJCC) 8th edition staging system for patients with hepatocellular carcinoma (HCC): a surveillance, epidemiology, end results (SEER) analysis. J. Surg. Oncol..

[12] Harding J.J., Nandakumar S., Armenia J. (2019). Prospective genotyping of hepatocellular carcinoma: clinical implications of next-generation sequencing for matching patients to targeted and immune therapies. Clin. Cancer Res..

[13] Greten T.F., Villanueva A., Korangy F. (2023). Biomarkers for immunotherapy of hepatocellular carcinoma. Nat. Rev. Clin. Oncol..

[14] Wang Z., Liu M., Zhang D.Z. (2022). Microwave ablation versus laparoscopic resection as first-line therapy for solitary 3-5-cm HCC. Hepatology.

[15] Xing R., Gao J., Cui Q. (2021). Strategies to improve the antitumor effect of immunotherapy for hepatocellular carcinoma. Front. Immunol..

[16] Cheng A.L., Qin S., Ikeda M. (2022). Updated efficacy and safety data from IMbrave150: atezolizumab plus bevacizumab vs. sorafenib for unresectable hepatocellular carcinoma. J. Hepatol..

[17] Sangro B., Chan S.L., Kelley R.K. (2024). Four-year overall survival update from the phase III HIMALAYA study of tremelimumab plus durvalumab in unresectable hepatocellular carcinoma. Ann. Oncol..

[18] Shen X., Zhao B. (2018). Efficacy of PD-1 or PD-L1 inhibitors and PD-L1 expression status in cancer: meta-analysis. BMJ (Clin. Res. ed).

[19] Rimassa L., Finn R.S., Sangro B. (2023). Combination immunotherapy for hepatocellular carcinoma. J. Hepatol..

[20] Ponvilawan B., Roth M.T. (2023). Sequencing systemic therapy in hepatocellular carcinoma. Curr. Treat. Options Oncol..

[21] Hertz D.L., Mcleod H.L. (2016). Integrated patient and tumor genetic testing for individualized cancer therapy. Clin. Pharmacol. Ther..

[22] Xing X., Hu E., Ouyang J. (2023). Integrated omics landscape of hepatocellular carcinoma suggests proteomic subtypes for precision therapy. Cell Reports Med..

